# Limited Discrepancy Between Cognitive Ability and Daily Living Skills in Autism: A Longitudinal Study From Ages 2–25

**DOI:** 10.1002/aur.70280

**Published:** 2026-06-02

**Authors:** Elaine B. Clarke, Catherine Lord, Vanessa H. Bal

**Affiliations:** ^1^ Graduate School of Applied and Professional Psychology Rutgers, The State University of New Jersey Piscataway New Jersey USA; ^2^ Department of Psychiatry and Biobehavioral Sciences University of California, Los Angeles Los Angeles California USA

**Keywords:** autism spectrum disorder, developmental disabilities, intelligence, latent variable modeling, longitudinal studies

## Abstract

Many autistic individuals with average or higher cognitive abilities (also referred to as intelligence quotient; IQ) exhibit weaker than expected daily living skills (DLS). However, existing evidence is primarily cross‐sectional. This study examined: (1) how IQ‐DLS discrepancies develop from early childhood through early adulthood, (2) whether childhood factors (e.g., autism features, non‐verbal IQ) predict these developmental pathways, and (3) how IQ‐DLS discrepancy trajectories relate to adult experiences (e.g., employment, well‐being, relationships). Analyses included 92 individuals from the Longitudinal Study of Autism with average or better IQ and repeated assessments of IQ and DLS from ages 2–25 years. Developmental patterns in IQ‐DLS discrepancy scores were identified using group‐based trajectory modeling. Two trajectory groups were identified: IQ = DLS (commensurate cognitive and daily living skills) and IQ > DLS (weaker DLS than expected for IQ). Most participants had commensurate IQ and DLS across development. When discrepancies emerged, they did so gradually, becoming apparent by mid‐childhood. White participants were more likely to be in the IQ > DLS group; participant race was the only early childhood predictor of group membership. IQ‐DLS discrepancy trajectories were not significantly associated with adult experiences. In contrast to existing literature, this study suggests autistic individuals with average or higher IQ may not always have persistent deficits in DLS relative to cognitive ability. When discrepancies occurred in this sample, they developed over time rather than being present from early childhood. These findings challenge deterministic assumptions about adaptive function in autism and highlight the importance of sustained opportunities to learn and practice DLS across development.

## Summary

In this study, we followed autistic people with average or higher cognitive ability from early childhood into adulthood to see how their cognitive ability and life skills developed together. Most participants had life skills that matched their cognitive abilities across development; for participants whose life skills fell behind, this gap appeared gradually in later childhood rather than early in life. These results differ from many earlier studies that have found autistic people with average or high cognitive ability have weaker life skills than expected and suggest that autistic people can develop life skills over time, especially when given opportunities to learn and practice.

## 1 Introduction

Daily living skills (DLS) are a predictor of adult autonomy and well‐being in autism spectrum disorder (ASD; referred to throughout as autism) and other developmental conditions (Laxman et al. 2019). Yet many autistic individuals experience persistent challenges in this area. A range of skills, from hygiene to time management, fall under the umbrella of DLS (Sparrow et al. 2016). Broadly speaking, DLS can be divided into Personal (e.g., toileting, bathing, and grooming), Domestic (e.g., cooking, household chores), and Community (e.g., obeying traffic signals, using money, ordering in a restaurant) skills. See Glover et al. (2022) for a detailed description of DLS subdomains.

DLS comprise one domain of the broader construct of adaptive function, which is defined as the collection of conceptual, social, and practical skills needed to independently navigate daily life (Floyd et al. 2015). Communication and socialization skills, in addition to DLS, are commonly conceptualized as domains of adaptive function (Sparrow et al. 2016). Prior work suggests that DLS are most directly linked to functional independence and adult experiences such as employment and independent living (Auld et al. 2022). DLS are also relatively distinct from other adaptive domains. Communication skills are closely tied to expressive and receptive language, which overlap with indices of cognitive ability, while socialization skills are closely associated with autism features (Clarke and Lord 2023). As such, discrepancies between cognitive ability and adaptive functioning are most clearly interpretable within the DLS domain. For these reasons, the current study focuses specifically on DLS.

Many autistic individuals with average or higher intellectual quotient (IQ) exhibit lower DLS than expected based on their estimated cognitive ability (i.e., IQ; Kanne et al. 2011). This pattern of comparatively strong IQ and weak DLS in autistic individuals (referred to throughout as IQ > DLS) was first described nearly two decades ago (Klin et al. 2007) and has been well‐documented since (Duncan and Bishop 2015; Kraper et al. 2017; McQuaid et al. 2021). In these studies, a minority of participants had similar IQ and DLS (referred to throughout as IQ = DLS). Despite robust evidence of IQ‐DLS discrepancies in autistic youth and adults, almost all studies are cross‐sectional. Thus, it is unclear how discrepancies emerge and change or remain stable over time. Understanding the relationship between IQ and DLS may provide important insights into timing and types of supports that could be offered in childhood and adolescence to promote autistic adult success.

### 1.1 Do IQ‐DLS Discrepancies Impact Autistic Adulthood?

Prior evidence suggests that DLS predict adult milestone attainment (e.g., paid employment, living independently) for autistic individuals with average or higher cognitive abilities (Gray et al. 2014; McCauley et al. 2020). Similarly, strong DLS are concurrently associated with low internalizing (Donoso et al. 2023; Gardiner and Grace 2018) and externalizing symptoms (Ashwood et al. 2015; Carpenter et al. 2022). Prior work in the same cohort used for the current study, the Longitudinal Study of Autism (LSA; Lord et al. 2006) found that childhood DLS levels predicted adult well‐being (Clarke and Lord 2025). Studies in samples of autistic individuals that include those with co‐occurring intellectual disability have found higher cognitive abilities in childhood are associated with more positive adult outcomes (Lord et al. 2020). Notably, several studies of autistic individuals with average or higher IQ have not found similar associations (Farley et al. 2009; Mason et al. 2021).

Longitudinal methods are necessary to determine how IQ‐DLS discrepancies emerge across development and change or remain stable with increasing age. Little work to date has considered whether early childhood characteristics (e.g., autism features) predict the likelihood of later IQ‐DLS discrepancies. It is also unclear how the presence of discrepancies impacts adult experiences. For example, smaller IQ‐DLS discrepancies in childhood may be associated with an increased likelihood of adult milestone attainment; little work to date has examined this possibility. It is also unknown whether IQ‐DLS discrepancies vary across specific types of DLS (e.g., Personal, Domestic, Community) in autistic individuals with average or higher IQ, and if so, whether this has implications for adulthood. A recent study of LSA cohort participants with autism and co‐occurring ID indicated that patterns of IQ‐DLS discrepancies differed in DLS subdomains compared to domain‐level scores, suggesting meaningful variability in growth patterns across DLS subdomains (Clarke et al. 2026). The current study is among the first to examine IQ‐DLS discrepancies by DLS subdomains in autistic individuals with average or better IQ.

## 2 The Current Study

This study leverages the LSA cohort to examine trajectories of IQ‐DLS discrepancies from 2 to 25 years in autistic individuals with average or higher IQ. Data were collected five times from 2 to 25, as described below. This study also examines whether early childhood characteristics (from approximately age 2) predict IQ‐DLS discrepancy trajectory inclusion, and whether trajectories are associated with adult experiences. The aims and hypotheses were as follows:

### 2.1 Aim 1

Calculate trajectories of IQ‐DLS discrepancies (using domain‐level standard scores and subdomain‐level age equivalents (AEs)) from ages 2–25 in a sample of autistic individuals with average or higher IQ. In line with prior research examining standard score discrepancies (Alvares et al. 2019; Duncan and Bishop 2015), it is hypothesized that the majority of participants will have IQ > DLS (i.e., IQ‐DLS standard scores discrepancies of one standard deviation or greater) while a minority of participants will have IQ = DLS. Given existing evidence suggesting IQ tends to increase across childhood in autistic individuals (Simonoff et al. 2020; Solomon et al. 2023), while DLS standard scores remain stable or decrease (Chen et al. 2023; Farmer et al. 2018), IQ‐DLS standard score discrepancies are expected to increase (i.e., IQ will be stronger than DLS) from early childhood through adulthood. Given little existing research, there were no a priori hypotheses regarding differences in IQ‐DLS AE discrepancy score trajectories by DLS subdomain.

### 2.2 Aim 2

Examine whether early childhood characteristics predict IQ‐DLS discrepancy group inclusion. Given prior cross‐sectional findings that more autism features and higher cognitive ability are associated with larger IQ‐DLS discrepancies (Duncan and Bishop 2015; Kanne et al. 2011; Kraper et al. 2017), it is hypothesized that higher levels of autism features and higher IQ in early childhood will be associated with inclusion in IQ > DLS trajectory groups across development.

### 2.3 Aim 3

Describe the phenotypic characteristics and adult experiences associated with inclusion in the identified IQ‐DLS discrepancy groups. It is hypothesized that higher IQ and lower levels of autism features will be associated with inclusion in IQ = DLS discrepancy trajectory groups across development. In line with prior work (Donoso et al. 2023; Wallace et al. 2016) it is expected that inclusion in IQ > DLS discrepancy trajectory groups will be associated with more internalizing and externalizing symptoms in adulthood, as well as decreased likelihood of adult milestone attainment (e.g., employment, living independently).

## 3 Method

### 3.1 Participants

Consecutive referrals (*N* = 213) under 37 months old to clinics in North Carolina and Chicago were enrolled in the Longitudinal Study of Autism. At age 9, 40 children from Michigan of similar age and diagnostic characteristics joined the cohort, resulting in 253 participants total. Detailed descriptions of this sample can be found in prior work (Lord et al. 2006). The present analyses included 92 participants with (1) a verbal IQ score of 70 or higher at age 9 and (2) data on IQ and DLS from at least two timepoints between ages 2–25 (M timepoints = 2.88, SD = 1.38). Prior work in this cohort (Anderson et al. 2013) and other longitudinal autism samples (Charman et al. 2011; Szatmari et al. 2009) indicate cognitive abilities are largely stable after mid‐ to late childhood.

### 3.2 Procedures

A battery of questionnaires and direct testing, including the Autism Diagnostic Observation Schedule (ADOS; Lord et al. 2000; Lord et al. 2012), IQ tests chosen from a standard hierarchy (Anderson et al. 2013), and the VABS (Sparrow et al. 1984; Sparrow et al. 2005) were completed at approximately ages 2, 3, 5, 9, 18, and 25. Adult milestone data was collected via survey packets completed by caregivers and participants capable of self‐report at approximately age 33. Ethical approval was obtained from the Institutional Review Boards at UCLA, University of Michigan, University of Chicago, and University of North Carolina, Chapel Hill, as appropriate. Parents and participants over 18 who were their own legal guardians gave written consent as required by the relevant institutional review board(s) prior to visits.

### 3.3 Measures

#### 3.3.1 Autism Features

The Autism Diagnostic Observation Schedule (ADOS; Lord et al. 2000; Lord et al. 2012) is a semi‐structured, clinician‐administered assessment measuring social communication impairments and restricted and repetitive behaviors (RRBs). The ADOS produces a total calibrated severity score (CSS; Gotham et al. 2009) and CSS for social affect (SA) and RRB (Hus et al. 2014). CSS ranges from 1 to 10; higher scores indicate more difficulties in social communication and/or clearer RRB during the ADOS. ADOS scores from age 9 are used to describe the sample (Table 1), as prior work indicates autism features are relatively stable after mid‐childhood (Elias and Lord 2022).

**TABLE 1:** Descriptive characteristics of the current sample by IQ‐DLS trajectory groups.

	DLS domain		Personal subdomain		Domestic subdomain		Community subdomain	
	IQ = DLS *n* = 57	IQ > DLS *n* = 35	IQ = DLS *n* = 69	IQ > DLS *n* = 23	IQ = DLS *n* = 65	IQ > DLS *n* = 27	IQ = DLS *n* = 61	IQ > DLS *n* = 31
Demographic characteristics								
Sex (% female)	12.2%	8.6%	11.6%	8.7%	10.8%	11.1%	11.5%	9.7%
Diagnostic status (% DD^a^)	35.1%	20.0%	34.8%	13.0%	33.9%	18.5%	34.4%	19.4%
Race								
% White	**63.2%**	**88.6%**	68.1%	87.0%	66.2%	88.9%	65.6%	87.1%
% Black/African American	**31.6%**	**5.7%**	27.5%	4.3%	29.2%	3.7%	31.1%	3.2%
% Asian/Pacific Islander	**1.8%**	**0%**	1.4%	0%	1.5%	0%	1.6%	0%
% American Indian	**1.8%**	**2.9%**	1.4%	4.3%	1.5%	3.7%	0%	6.5%
% Multiracial	**1.8%**	**2.9%**	1.4%	4.3%	1.5%	3.7%	1.6%	3.2%
Ethnicity (% Hispanic/Latino)	3.5%	8.6%	2.9%	13.0%	3.1%	11.1%	3.3%	9.7%
Urbanicity (% urban)	33.3%	34.3%	33.3%	34.8%	32.3%	37.0%	34.4%	32.3%
Caregiver education (% college education)	35.1%	54.3%	42.0%	43.5%	38.5%	51.9%	34.4%	58.1%
Phenotypic characteristics at age 2 M (SD)								
Verbal IQ	66.39 (31.04)	65.06 (26.67)	66.87 (30.08)	62.91 (27.30)	66.28 (29.80)	64.93 (28.63)	63.92 (28.77)	69.74 (30.46)
Nonverbal IQ	86.23 (17.74)	88.91 (17.71)	86.13 (16.80)	90.61 (20.12)	85.25 (16.64)	92.07 (19.46)	83.90 (16.71)	93.84 (17.95)
Vineland ABC^b^	69.16 (9.38)	67.76 (8.24)	69.52 (9.51)	66.20 (6.73)	69.80 (9.59)	65.86 (6.54)	69.76 (9.49)	65.95 (6.92)
ADOS CSS^c^	4.50 (2.98)	5.34 (2.99)	4.66 (2.91)	5.30 (3.26)	4.73 (2.96)	5.03 (3.14)	4.62 (2.95)	5.22 (3.10)
ADOS CSS—SA^d^	4.98 (3.13)	5.62 (2.77)	5.10 (3.04)	5.60 (2.90)	5.18 (3.04)	5.33 (2.94)	5.06 (3.01)	5.54 (2.99)
ADOS CSS—RRB^e^	4.33 (2.59)	4.80 (2.85)	4.43 (2.58)	4.73 (3.04)	4.50 (2.59)	4.51 (2.95)	4.40 (2.66)	4.70 (2.77)
Phenotypic characteristics at age 9 M (SD)								
Verbal IQ	**93.37** **(17.48)**	**105.37** **(19.79)**	96.64 (19.17)	101.83 (19.17)	96.05 (19.40)	102.48 (18.25)	94.80 (18.85)	104.10 (18.66)
Nonverbal IQ	93.45 (16.47)	104.54 (18.89)	93.03 (16.81)	108.65 (16.94)	93.31 (16.38)	107.43 (18.38)	92.17 (15.89)	109.38 (17.35)
Vineland ABC^b^	82.97 (19.86)	76.85 (19.99)	81.51 (20.95)	77.67 (18.39)	83.58 (21.08)	74.45 (17.01)	81.67 (20.86)	77.59 (18.64)
ADOS CSS^c^	3.63 (2.65)	4.94 (2.88)	3.78 (2.75)	5.17 (2.74)	3.66 (2.72)	5.26 (2.71)	3.62 (2.71)	5.13 (2.74)
ADOS CSS—SA^d^	3.91 (2.65)	4.97 (2.91)	4.04 (2.71)	5.13 (2.88)	3.94 (2.71)	5.22 (2.79)	3.84 (2.68)	5.26 (2.78)
ADOS CSS—RRB^e^	4.84 (2.86)	5.54 (3.28)	4.84 (2.96)	5.91 (3.13)	4.85 (2.90)	5.74 (3.29)	4.89 (2.95)	5.55 (3.17)

*Note:* Text in bold indicates significant group differences at *p* ≤ 0.01 for the IQ‐DLS SS trajectory groups. Statistical comparisons were not conducted for the AE trajectory groups, given considerable similarities in growth patterns across the DLS domain and Personal, Domestic, and Community subdomains.

^a^ Non‐spectrum developmental disability.

^b^ Adaptive behavior composite.

^c^ Autism Diagnostic observation schedule, calibrated severity score.

^d^ Social affect.

^e^ Restricted, repetitive behaviors.

Twenty‐six participants in the current study were never diagnosed with ASD across repeated assessments. Those with and without ASD diagnoses in this cohort share many similarities across development (Lord et al. 2020). Thus, participants who never received an ASD diagnosis were retained in the current analyses. The diagnostic status (autism or non‐spectrum developmental delay) of participants within each IQ‐DLS discrepancy trajectory group is reported in Table 1.

#### 3.3.2 Adult Milestones

##### 3.3.2.1 Employment, Education, Living Status, and Relationships

Data from self‐ and caregiver‐report demographic forms from approximately age 33 were used to create binary variables. Participant self‐report data was prioritized, and caregiver‐report data was only used when self‐report was unavailable. For employment, 1 indicated the participant was currently working for pay in the community either full or part‐time, and 0 indicated the participant was either unemployed or working without pay (e.g., volunteering). For education, 1 indicated the participant had completed at least a four‐year college degree, and 0 indicated the participant had not completed a four‐year college. For living status, 1 indicated the participant lived independently and 0 indicated the participant lived in the family home or a supported setting. For social relationships, 1 indicated at least one mutual friendship and 0 indicated no mutual friendships. Finally, for romantic relationships, 1 indicated the participant was currently or had previously been in a romantic relationship, 0 indicated the participant had never been in a romantic relationship.

##### 3.3.2.2 Well‐Being

Happiness quotient composite scores (McCauley et al. 2020) were used to measure well‐being. Scores range from −1 to 1, with higher scores indicating greater reported levels of subjective well‐being. Self‐report happiness quotient scores were only available for a portion of the current sample (*n* = 40). To improve analytic power, in cases where self‐report scores were unavailable, caregiver‐report happiness quotient scores were also used in analyses (*n* = 29). Prior work shows that self‐ and caregiver‐report happiness quotient scores in this sample are strongly positively correlated (McCauley et al. 2020).

##### 3.3.2.3 Internalizing and Externalizing Behaviors

Caregivers were asked to complete questionnaires on participants' internalizing and externalizing symptoms in adulthood. Internalizing was measured using the Internalizing subscale of the Adult Behavior Checklist (ABCL; Achenbach and Rescorla 2003). Externalizing was measured using the externalizing subscale of the ABCL. ABCL data were collected at approximately age 26 (*m*_age_ = 26.38, SD = 3.21).

#### 3.3.3 Cognitive Ability

The Mullen Scales of Early Learning (MSEL; Mullen 1995) were administered at age 2. Later cognitive assessments were chosen from a standard hierarchy including the Wechsler Intelligence Scale for Children (WISC; Wechsler 2003, 2011), Wechsler Abbreviated Scale of Intelligence (WASI; Wechsler 1999), Differential Abilities Scale (DAS; Elliott 2007), and MSEL. AEs from nonverbal domain subtests were averaged to calculate nonverbal mental age, herein referred to as NV abilities. As detailed below (see *IQ‐DLS Discrepancy Scores*), SS were used in DLS domain analyses, and AEs were used in subdomain (i.e., personal, domestic, community skills) analyses.

#### 3.3.4 DLS

The Vineland Adaptive Behavior Scales (VABS; Sparrow et al. 1984; Sparrow et al. 2005) comprehensive clinician‐interview form administered to a caregiver was used to assess participants' adaptive functioning. Participants completed the first edition of the VABS (Sparrow et al. 1984) from ages 2–9 years and the second edition (Sparrow et al. 2005) from ages 14–26 years. For individuals over the age of 7, the VABS assesses adaptive behaviors in three primary domains: Communication, DLS, and Socialization. VABS Adaptive Behavior Composite (ABC) standard scores from age 9 are used to describe the current sample (Table 1).

The DLS domain of the VABS comprises three subdomains: Personal, Domestic, and Community. The Personal subdomain assesses DLS related to self‐care, including dressing, bathing, and physical health. The Domestic subdomain assesses DLS related to household maintenance, including chores, meal preparation, and laundry. The Community subdomain assesses DLS related to navigating public spaces, including making purchases, running errands, and using public transportation. Across both VABS and VABS‐II, an AE estimate can be derived from each subdomain. AEs from each of the DLS subdomains were used to calculate separate IQ‐DLS discrepancy scores for Personal, Domestic, and Community skills.

#### 3.3.5 IQ‐DLS Discrepancy Scores

Four IQ‐DLS discrepancies were calculated for each timepoint, one for the DLS domain, and one each for the Personal, Domestic, and Community subdomains. Positive discrepancy scores indicate DLS are lower than expected based on cognitive abilities (IQ > DLS) and scores close to zero indicate comparable DLS and cognitive abilities (IQ = DLS; see Clarke et al. 2026 for a detailed description).

IQ‐DLS domain discrepancies were calculated by subtracting VABS DLS standard scores from full‐scale IQ standard scores. In recognition of the fact that, in contrast to cognitive abilities, adaptive skills that exceed age‐expectations may be cause for concern (see Dariotis et al. 2023), and in line with the approach described by Duncan and Bishop (2015), a discrepancy score of greater than ±15 points (i.e., one standard deviation) would indicate the presence of a significant difference between DLS and IQ standard scores. To avoid penalizing those with above‐average IQ in the calculation of IQ‐DLS standard score discrepancies, full‐scale IQ scores greater than 100 were transformed to equal 100 for IQ‐DLS discrepancy calculations. This ensured that for individuals with above‐average IQ and adequate DLS (e.g., full‐scale IQ of 115 and DLS standard score of 90), discrepancy scores remained within ±15 points (e.g., 100–90 = 10 points vs. 115–90 = 25 points, which would falsely indicate a deficit in DLS; Duncan and Bishop 2015).

Subdomain v‐scale (i.e., standard) scores were not introduced until the second edition of the VABS (Sparrow et al. 2005). Participants in the current study completed the first edition of the VABS from ages 2–9, thus discrepancies between IQ and DLS standard scores could not be calculated at the subdomain level across development. Personal, Domestic, and Community subdomain IQ‐DLS discrepancies were calculated by subtracting subdomain AEs from NV abilities (i.e., averaged AEs from nonverbal subtests). See Clarke et al. (2026) for a detailed description of this approach.

### 3.4 Data Analysis

#### 3.4.1 Aim 1

Group‐based trajectory modeling was performed using the traj plugin in Stata 16 (Jones and Nagin 2007, 2013). Group‐based trajectory modeling estimates developmental trajectories via maximum likelihood estimation. The best‐fitting model (linear, quadratic, etc.) and the number of trajectory groups were determined using the Bayesian Information Criterion (BIC). Unconditional 1, 2, and 3 class models were compared using BIC and the smallest group membership percentage (Table 2). After classes were determined, higher‐order effects were tested to establish whether cubic, quadratic, linear, or intercept modeling best explained variation over time.

**TABLE 2:** IQ‐DLS standard score and age equivalent discrepancy trajectories model selection (*n* = 92).

Model	DLS domain standard scores			Personal subdomain age equivalents		
	BIC	Model	BIC	BIC	AIC	Smallest group %
1 Class model	−1797.53	1 Class model	−1797.53	−1935.83	−1932.08	—
2 Class model	**−1772.99**	2 Class model	**−1772.99**	**−1899.58**	**−1892.09**	**34.12**
3 Class model	−1771.46	3 Class model	−1771.46	−1906.33	−1895.09	0.01

	Domestic subdomain age equivalents			Community subdomain age equivalents		
Model	BIC	AIC	Smallest group %	BIC	AIC	Smallest group %
1 Class model	−1880.07	−1876.34	—	−1820.93	−1817.18	—
2 Class model	**−1818.47**	**−1811.00**	**34.71**	**−1749.59**	**−1742.09**	**39.81**
3 Class model	−1822.62	−1811.42	1.10	−1752.47	−1741.22	1.10

*Note:* Group‐based trajectory modeling is a maximum likelihood estimation (MLE) technique, and thus does not produce *p*‐values. As noted in the text, bolded values indicate which model had the best fit statistics, as indicated by AIC, BIC, and smallest group %. AIC = Aikake Information Criterion, BIC = Bayesian Information Criterion.

#### 3.4.2 Aim 2

Binary logistic regression was used to examine early childhood predictors (i.e., ADOS CSS, nonverbal IQ at age 2) of IQ‐DLS standard score trajectory group inclusion, controlling for race and maternal education. Because these analyses were exploratory and intended to identify early predictors of later IQ‐DLS discrepancy trajectories, the *p* value for significant findings was set at 0.05.

#### 3.4.3 Aim 3

One‐way ANOVA and chi‐square analyses were used to compare IQ‐DLS standard score trajectory groups on demographic and phenotypic characteristics (i.e., biological sex, race, caregiver education, urbanicity, and ADOS CSS, IQ, and VABS ABC scores from age 9; Table 1) and adult outcomes (i.e., employment, education, living status, social and romantic relationships, well‐being, and ABCL scores; Table 3). To correct for multiple comparisons, the *p* value for significant findings was set at 0.01.

**TABLE 3:** Adult cognitive ability, milestones, and mental health characteristics of DLS domain trajectory groups.

Adult characteristics	Trajectory groups	
	IQ = DLS *n* = 57	IQ > DLS *n* = 35
Full‐Scale IQ M (SD)	97.40 (22.19)	120.0 (12.90)
Employment (% ever employed)	87.8%	78.6%
Education (% college education)	51.4%	38.1%
Living status (% living independently)	57.1%	50.0%
Friends (% with friends)	81.5%	67.9%
Romantic relationships (% who have dated)	68.7%	41.7%
Happiness factor score M (SD)	0.15 (0.74)	−0.04 (0.80)
ABCL^a^ externalizing M (SD)	49.93 (11.33)	47.72 (9.59)
ABCL^a^ internalizing M (SD)	56.00 (11.72)	57.12 (10.80)

*Note:* After corrections for multiple comparisons, there were no significant differences in adult characteristics by trajectory groups.

^a^ Adult Behavior Checklist (ABCL; Achenbach and Rescorla 2003).

### 3.5 Power Analyses

An a priori power analysis was conducted using G*Power version 3.1.9.7 (Faul et al. 2007) to determine the minimum sample size required for Aim 3 comparisons. Results indicated that the sample had 80% power to detect medium‐to‐large effects for planned group comparisons. Specifically, for two‐tailed independent‐samples comparisons, the minimum detectable effect size was approximately *d* ≥ 0.69 at a significance criterion of *α* = 0.01. For binary outcomes evaluated via chi‐square, the study had 80% power to detect effects of approximately *w* ≥ 0.33 at *α* = 0.01. In short, the current sample size was sufficient to detect medium‐to‐large effects but was underpowered to detect small effects across outcomes.

## 4 Results

### 4.1 Aim 1: IQ‐DLS Discrepancy Trajectories

#### 4.1.1 Standard Score Discrepancy Trajectories

**FIGURE 1:**
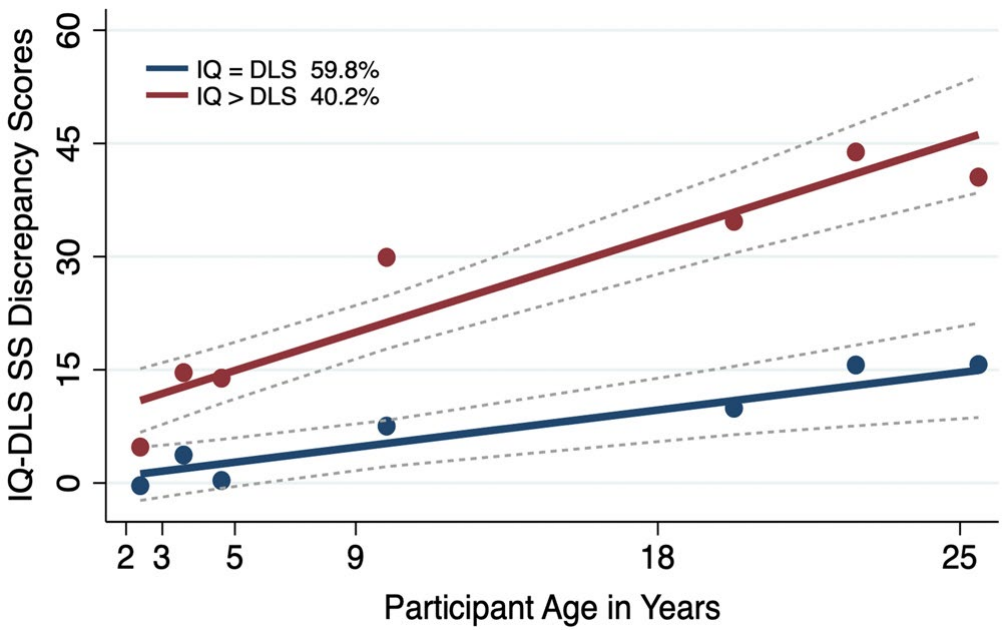
Trajectories of IQ‐DLS standard score discrepancies from ages 2–25.

A two‐group model best fit the data (Table 2). The slope of both groups was linear (Figure 1). Over half (57.4%) of participants fell in the “IQ = DLS” group, with IQ‐DLS discrepancies within one standard deviation (i.e., between −15 and 15), indicating relatively commensurate DLS and cognitive abilities from ages 2–25 (Table S1). Notably, as shown in Table 4, average discrepancies for the IQ = DLS group did increase slightly with increasing age, with the largest increases in discrepancies between ages 18–25, such that by age 25, IQ standard scores were close to one full standard deviation higher than DLS standard scores. The remaining 42.6% of participants were in the “IQ > DLS” group, which had higher IQ‐DLS discrepancies across development, on average over one standard deviation (i.e., greater than 15) for most of the study. From ages 2–5, the discrepancies of the IQ > DLS group were higher than those of the IQ = DLS group, but still within one standard deviation. From ages 9–25, the IQ > DLS group's average discrepancies were between one and two standard deviations, indicating a sizeable weakness in DLS compared to cognitive abilities.

**TABLE 4:** Average IQ‐DLS discrepancy score by timepoint and DLS domain and subdomain trajectory groups.

	Data collection timepoint						
	2	3	5	9	18	21	25
*n*	73	60	45	58	54	10	30
DLS domain M (SD) [range]							
IQ > DLS	4.0 (10.3) [−13.0–20.0]	15.1 (9.5) [−5.0–35.5]	14.7 (16.4) [−7.5–51.0]	31.6 (22.0) [−7.0–105.0]	36.9 (13.6) [19.0–69.0]	45.4 (8.0) [34.0–54.0]	43.8 (11.3) [25.0–64.0]
IQ = DLS	0.2 (14.7) [−27.0–30.0]	3.3 (14.2) [−28.5–35.5]	−0.6 (14.3) [−30.5–20.5]	5.5 (21.0) [−40.0–56.0]	9.5 (11.8) [−14.0–32.0]	15.0 (11.1) [4.0–27.0]	14.7 (8.0) [1.0–26.0]
Personal subdomain M (SD) [range]							
IQ > DLS	0.21 (0.46) [−0.55–0.98]	0.70 (0.69) [−0.18–2.67]	0.66 (0.78) [−0.37–2.02]	3.10 (1.91) [−1.03–6.12]	9.10 (3.79) [4.17–16.70]	11.03 (3.73) [5.62–14.09]	15.51 (2.78) [12.47–20.58]
IQ = DLS	0.01 (0.42) [−1.00–0.71]	0.06 (0.07) [−0.03–0.17]	0.20 (0.92) [−2.32–1.33]	1.31 (2.55) [−6.79–5.25]	2.99 (3.66) [−6.26–9.42]	5.14 (1.80) [2.84–7.84]	8.91 (2.96) [3.57–14.73]
Domestic subdomain M (SD) [range]							
IQ > DLS	0.28 (0.43) [−0.38–1.17]	1.15 (0.95) [0.33–4.42]	0.47 (0.72) [−0.30–2.18]	2.92 (1.78) [−0.28–5.77]	7.28 (4.53) [−1.15–17.62]	13.23 (2.68) [9.70–16.13]	14.88 (4.72) [5.93–22.50]
IQ = DLS	0.03 (0.38) [−1.02–1.03]	0.57 (0.80) [−1.47–2.62]	−0.08 (0.87) [−1.90–1.30]	0.78 (1.93) [−3.64–4.45]	1.16 (3.65) [−7.58–7.92]	6.59 (3.08) [3.05–8.71]	6.97 (3.23) [1.62–11.36]
Community subdomain M (SD) [range]							
IQ > DLS	0.58 (0.56) [−0.47–1.37]	1.37 (0.84) [0.28–3.83]	1.02 (0.74) [0.17–2.52]	1.58 (1.78) [−1.83–4.68]	7.16 (2.92) [2.95–14.45]	8.78 (3.08) [3.03–12.38]	11.11 (2.64) [4.93–14.13]
IQ = DLS	0.42 (0.51) [−0.69–1.41]	0.76 (0.61) [−0.88–1.93]	0.33 (0.79) [−1.71–1.40]	0.28 (2.18) [−5.85–5.20]	1.86 (2.78) [−3.33–12.25]	3.20 (1.61) [1.34–4.21]	4.74 (2.63) [0.82–8.42]

*Note:* IQ‐DLS AE discrepancy scores are presented in years. Positive IQ‐DLS discrepancy scores indicate cognitive abilities are higher than DLS (IQ > DLS). IQ‐DLS discrepancy scores of zero or close to zero indicate cognitive abilities and DLS are equal (IQ = DLS). Table S1 presents the mean, standard deviation, and range of full‐scale IQ and DLS domain standard scores by DLS domain trajectory groups. Tables S2–S4 present the mean, standard deviation, and range of NV abilities and DLS AEs by DLS subdomain trajectory groups.

**FIGURE 2:**
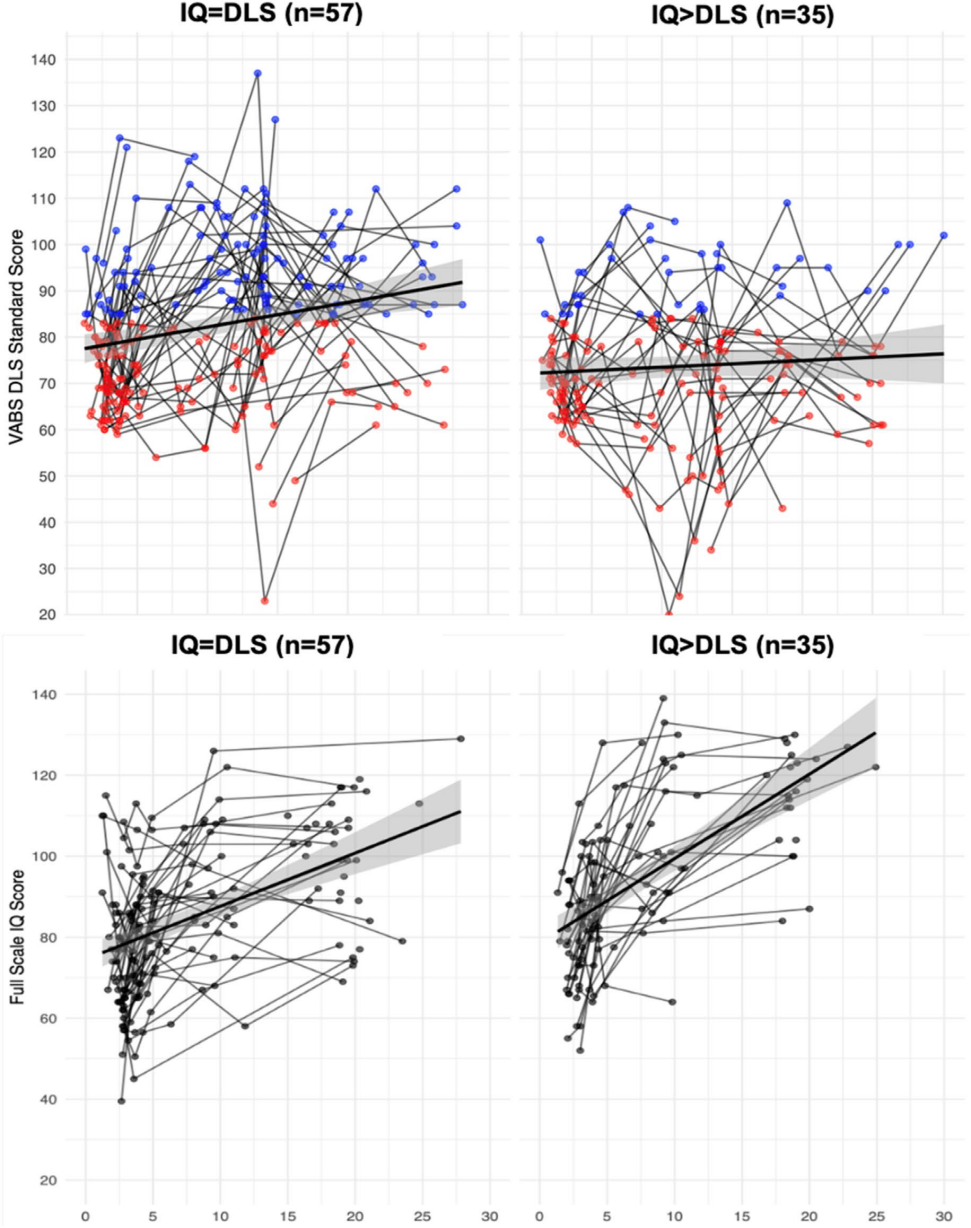
Individual growth patterns in IQ and DLS SS by IQ‐DLS SS discrepancy trajectory groups from ages 2–25.Blue dots indicate average or higher VABS DLS SS, red dots indicate lower than average VABS DLS SS.

Figure 2 descriptively characterizes growth patterns in IQ and DLS SS in this sample from ages 2–25. Participants' individual growth patterns across DLS domain standard scores and Personal, Domestic, and Community subdomain AEs were very similar; 71.7% of participants were in the same trajectory group (i.e., IQ = DLS or IQ > DLS) for the DLS domain and all three subdomains, 23.9% were in the same trajectory group for three out of the four, and 4.4% had variable trajectory group inclusion. Growth patterns were comparable when only autistic participants were included in the analyses (Table S5; Figures S1–S2).

#### 4.1.2 AE Discrepancy Trajectories

**FIGURE 3:**
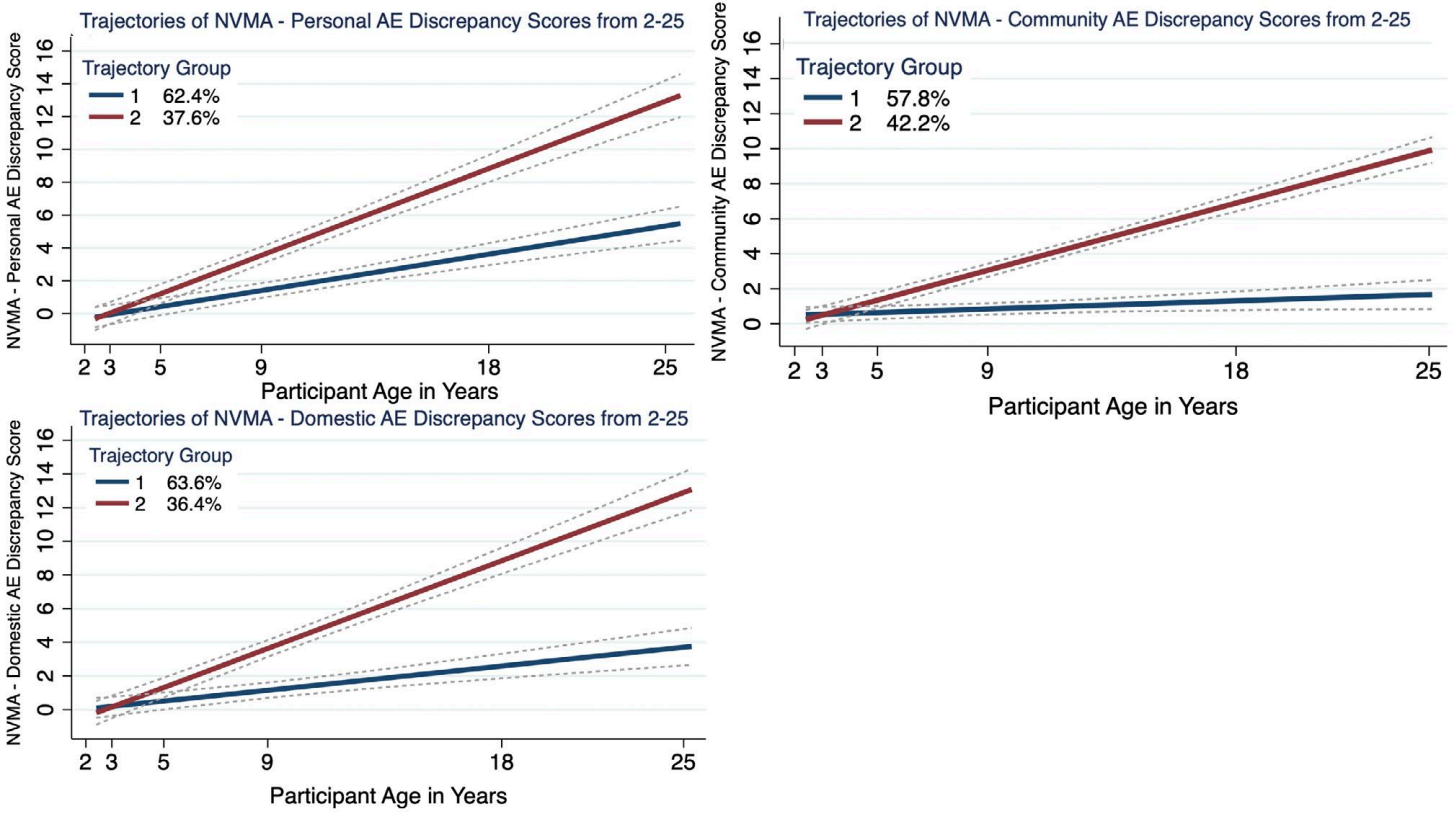
Trajectories of NVMA‐DLS AE discrepancies from ages 2–25.AE discrepancy scores are presented in years.

For all three DLS subdomains (Personal, Domestic, Community), a two‐group model best fit the data (Table 2). In each subdomain, approximately two‐thirds of participants fell in the IQ = DLS group, with commensurate NV abilities and DLS AEs across time, as indicated by IQ‐DLS discrepancy scores of zero or near‐zero. The remaining participants had IQ > DLS, with positive discrepancies that increased in magnitude with increasing age (Table 4), reflecting DLS AEs that increasingly lagged behind NV abilities across development (Tables S2–S4). These groups comprised anywhere from 34% to 40% of the sample for each subdomain (Figure 3).

### 4.2 Aim 2: Early Childhood Characteristics Associated With IQ‐DLS Discrepancy Trajectory Group Inclusion

Binary logistic regression was used to assess the predictive utility of early childhood characteristics on trajectory group inclusion. Given the similarity in group membership and developmental patterns across the DLS domain and subdomain trajectories, regression models were run for standard score IQ‐DLS trajectories only. Participant race and maternal education were entered in the first block. ADOS CSS and nonverbal IQ at age 2 were entered in the second block. The regression model is summarized in Table 5.

**TABLE 5:** Binary logistic regression model for IQ‐DLS domain standard score discrepancy trajectory groups.

	Variables entered	Coefficient	*p*	Odds ratio	95% CI	Block statistic	Overall model statistic
Block 1	Race	−1.18	0.**02**	0.307	0.08–1.06	***X*** ^***2***^ **(2) = 6.60, *p = 0*.037**	*X* ^*2*^(4) = 8.42, *p* = 0.07
	Maternal education	0.41	0.09	1.52	0.59–3.87		
	Non‐verbal IQ	0.005	0.75	1.00	0.97–1.03		
Block 2	ADOS total CSS^a^	0.114	0.19	1.12	0.94–1.32	*X* ^*2*^(2) = 1.81, *p* = 0.403	

*Note:* Text in bold indicates significant model block statistics at *p* < 0.05. All predictors entered into Block 2 were collected at approximately age 2.

^a^ Autism diagnostic observation schedule, calibrated severity score.

The first block of the model was significant (X^2^ (2, *n* = 87) = 6.60, *p* = 0.037). The model correctly classified 69.0% of cases. Participant race was a significant predictor of standard score IQ‐DLS discrepancy trajectory group membership (*p* = 0.020), but maternal education was not (*p* = 0.097). The second block, which included nonverbal IQ and ADOS CSS scores from age 2, did not significantly improve the model (X^2^ (2, *n* = 87) = 1.81, *p* = 0.403; Table 5).

### 4.3 Aim 3: Characteristics Associated With IQ‐DLS Discrepancy Trajectory Groups

Again, given the similarity in group membership and developmental patterns across the DLS domain and subdomain trajectories, descriptive comparisons were conducted for standard score IQ‐DLS trajectories only. There was a significantly higher proportion of white participants in the IQ > DLS trajectory group than the IQ = DLS group (*p* = 0.008). Participants in the IQ > DLS also had significantly higher verbal IQ scores at age 9 than participants in the IQ = DLS group (*p* = 0.006). Non‐verbal IQ scores (*p* = 0.021), Vineland ABC standard scores (*p* = 0.248) and ADOS SA (*p* = 0.076), RRB (*p* = 0.283), and total CSS (*p* = 0.028) from age 9 did not significantly differ across standard score IQ‐DLS trajectory groups. Participant sex (*p* = 0.579), ethnicity (*p* = 0.298), urbanicity (*p* = 0.925), maternal education (*p* = 0.097), and diagnostic status (*p* = 0.123) did not significantly differ across standard score trajectory groups (all *p* > 0.01). Finally, adult employment (*p* = 0.828), educational attainment (*p* = 0.333), residential status (*p* = 0.224), friendship status (*p* = 0.416), romantic status (*p* = 0.043), happiness factor scores (*p* = 0.293), ABCL externalizing scores (*p* = 0.433), and ABCL internalizing scores (*p* = 0.711) did not significantly differ by standard score IQ‐DLS trajectory groups (Table 3).

## 5 Discussion

A central contribution of the present study is the longitudinal examination of IQ and DLS as they relate to one another from early childhood to adulthood. Much of the existing literature on IQ‐DLS discrepancies relies on cross‐sectional data, which necessarily conflates developmental change with between‐person differences. By contrast, the current findings illustrate that IQ and DLS do not diverge uniformly across development. In recent years, a growing body of cross‐sectional work has suggested that deficits in DLS relative to cognitive ability are a common characteristic in autistic children (Bradshaw et al. 2019; Tillmann et al. 2019) and adults (Alvares et al. 2019) with average or better IQ. Work by McQuaid et al. (2021) suggested that this gap between cognitive ability and adaptive functioning may increase with age. Mirroring this, the subset of participants in the IQ > DLS group in the current study showed increasing divergence over time. Yet the majority (57.9%) of participants in the current study had commensurate IQ and DLS from ages 2–25 (as indicated by standard scores within one standard deviation). These findings underscore the importance of longitudinal approaches in distinguishing stable individual differences from developmental divergence in IQ and DLS.

These findings also raise concerns about the common assumption that autistic individuals with average or higher IQ will inevitably exhibit deficits in DLS. For the subset of participants in this study whose DLS were substantially weaker than their cognitive abilities, this discrepancy emerged gradually and was not apparent until mid‐to‐late childhood. This may indicate that IQ‐DLS discrepancies are not a fixed outcome for autistic individuals. Rather, IQ‐DLS discrepancies may reflect differences in expectations for DLS or opportunities to learn and practice DLS over time (Teh et al. 2024).

From this perspective, assuming early competence in the ability to learn DLS—and providing consistent, developmentally appropriate opportunities to learn and practice—may be particularly important for autistic individuals. In line with this, a recent randomized control trial found that autistic adolescents with average or higher cognitive abilities enrolled in an intervention designed to improve DLS made significant improvements in Personal and Domestic (but not Community) DLS compared to an active control group (Duncan et al. 2025). This suggests that autistic individuals can learn and use DLS with targeted teaching and support. Deficit‐oriented assumptions may inadvertently limit opportunities for DLS acquisition in autistic youth who otherwise demonstrate the capacity to learn and use DLS.

The consistency of the trajectory structure across the DLS domain and the Personal, Domestic, and Community subdomains in this study is striking. In all four cases, two linear trajectory groups were identified: an IQ = DLS group and an IQ > DLS group (Figures 1, 2, 3). This remained the case when only autistic participants were included in analyses (Figures S1–S2). This contrasts with prior analyses in the LSA cohort of autistic individuals with co‐occurring intellectual disability (ID; Clarke et al. 2026). In that study, two trajectory groups were identified at the DLS domain‐level, but three distinct trajectory groups emerged for the Personal, Domestic, and Community subdomains, reflecting greater heterogeneity in how adaptive skills differed from cognitive abilities across DLS subdomains. This may suggest that the relationship between cognitive and adaptive abilities is more consistent across DLS subdomains for autistic individuals with average or higher IQ, whereas subdomain‐specific differences may be more common for autistic individuals with co‐occurring ID. Further work in independent samples is needed to better understand how the presence of ID shapes associations between cognitive ability and DLS.

A significantly higher percentage of white participants were in the IQ > DLS standard score trajectory group, compared to the IQ = DLS group. Similar trends for race were seen in the Domestic and Community AEs trajectory groups. Regression analyses indicated that participant race was the only characteristic from early childhood that predicted IQ‐DLS standard score trajectory group inclusion. Several studies in independent samples of autistic children have found that white individuals tend to have lower scores on measures of adaptive behavior than children of color (Furnier et al. 2024; Ratto et al. 2016) and higher scores on measures of cognitive ability (Maenner 2023). In line with this, a post hoc analysis of the current sample indicated that White participants had significantly higher IQ scores than Black participants (*t*(90) = 3.61, *p* < 0.001). This may have contributed to the observed racial differences in trajectory group membership, as participants with relatively higher IQ scores may have been more likely to have DLS that fell below expectations based on cognitive ability. However, because IQ values above 100 were transformed for IQ‐DLS discrepancy calculations to avoid inflating discrepancies for those with above‐average cognitive abilities, this is unlikely to fully explain the pattern observed here. Further, DLS domain standard scores for the IQ = DLS group were an average of 6.92 points higher than standard scores for the IQ > DLS group across development (Table S1), indicating the difference between trajectory groups was not driven solely by differences in IQ scores.

In short, the present analyses do not provide insight into why white participants had higher IQ‐DLS discrepancy scores than participants of color (predominantly Black/African American participants in the current sample; see Table 1). As suggested by Ratto et al. (2016), one possibility is that caregivers from racial/ethnic minority groups may underreport challenges in DLS. In line with this, prior analyses of the LSA cohort have indicated that white caregivers report increased burden associated with caring for an autistic individual than caregivers of color, despite few to no differences in child phenotypic characteristics (e.g., cognitive ability, autism features, etc.) by race/ethnicity (Carr and Lord 2013; Christopher et al. 2023). The current findings likely reflect a combination of measurement factors and broader contextual influences.

Another possibility is that expectations for DLS and opportunities for DLS learning and practice may meaningfully differ across racial/ethnic groups. There is considerable evidence from the general population that children of color and children from less advantaged socioeconomic backgrounds experience higher familial expectations for DLS competence than white children, including assisting in more household tasks and having greater independence in such tasks at earlier ages (Burton 2007; Hafford 2010; Weisner 1987). Given that DLS was measured via caregiver report, it is impossible to know how much the racial‐ethnic differences in IQ‐DLS discrepancy scores seen here were driven by differences in how the items were rated versus actual differences in the DLS of participants of varying racial/ethnic backgrounds. Future qualitative and mixed methods work examining how caregivers from diverse racial/ethnic backgrounds understand, value, and report DLS may clarify the mechanisms underlying these observed differences.

### 5.1 Implications for Autistic Adulthood

When young children receive autism diagnoses, families often ask questions about the future: “How likely is my child to live on their own? Will my child ever go to college or get married?” Of course, such questions are difficult to answer for any young child. Nonetheless, the current findings underscore the particular challenge of answering such questions in the context of autism. In this sample of early‐identified autistic individuals with average or higher cognitive abilities, there is considerable variability in the achievement of adult milestones. In contrast to our Aim 3 hypothesis, this variability does not appear to be associated with the relative size of the discrepancy between cognitive abilities and DLS across development.

Notably, participants in the IQ > DLS group had higher cognitive ability in adulthood than those in the IQ = DLS group (Table 3). One possible explanation for the lack of difference in adult milestones is that this relative strength in cognitive ability may allow individuals to compensate for relative weaknesses in DLS in certain contexts (e.g., through abstract reasoning skills), thereby reducing observable differences in broad outcomes such as employment or living status. As such, the absence of group differences should not be interpreted to indicate that discrepancies between cognitive ability and DLS are inconsequential, but rather that their impact may depend on the context in which skills are required.

Power analyses indicated that the study was sufficiently powered to detect medium‐to‐large effects but underpowered to reliably identify smaller associations. As a result, current null findings should be interpreted with caution, as the absence of statistically significant differences in adult milestones by IQ‐DLS trajectory groups does not necessarily indicate the absence of meaningful associations between these constructs.

The current null results should not be interpreted to indicate that IQ and DLS (and the relative difference between these constructs) are irrelevant to adult functioning. There is a wealth of evidence to illustrate the importance of both DLS and cognitive abilities in supporting the success of autistic adults (Farley et al. 2018; Pickles et al. 2020; Smith et al. 2012). Instead, these results speak to the complexity of adult life and of understanding predictors of broad “outcomes” such as employment. In the present study, participants were categorized based on whether or not they reported paid employment, regardless of the number of hours they were working. Indeed, beyond part‐time and paid jobs, employment itself encompasses a wide range of roles with distinct cognitive, adaptive, and social demands. As in the general population, no single characteristic or handful of characteristics can predict milestone attainment for autistic individuals. Given the absence of strong associations between IQ–DLS discrepancy trajectories and adult milestones in this study, clinicians should *not* interpret early discrepancies to make deterministic predictions about adult outcomes. Rather, the current results support clinical focus on ongoing assessment and support for DLS across development.

### 5.2 Limitations

Ascertained in the early 1990s, at a time when autism awareness was less prevalent, the LSA cohort consists of a unique group of adults with autism and related developmental conditions. These participants may be different from both currently diagnosed younger individuals and same‐aged individuals diagnosed later in development (Lord et al. 2022). There are also relatively few female participants in this sample, constraining the ability to test for sex‐based differences. While attrition in this longitudinal cohort is comparable to other population‐based (Gustavson et al. 2012) and autism‐specific (Taylor and Mailick 2014) longitudinal samples, missing data, particularly at later time points, may have impacted these findings. Compared to white participants, a higher proportion of participants from minority racial/ethnic backgrounds have been lost to follow‐up during the three decades since the cohort's inception. Additionally, data on service receipt in this cohort are limited, which constrained our ability to examine whether engagement in intervention was associated with these trajectory groups.

Some of the characteristics that make the LSA cohort unusual also provide an important comparison point to recent studies of autistic adults with average or higher cognitive abilities. Specifically, such studies often rely exclusively on survey data, cannot independently verify autism diagnoses, and include samples that are largely white, urban, well‐educated, and contain a higher proportion of biological females. In contrast, the LSA cohort is comprised of individuals with confirmed childhood diagnoses of autism and related developmental conditions and includes a relatively high proportion of participants from minority racial/ethnic groups, as well as those from rural areas and low socioeconomic backgrounds. Together, these differences in cohort composition suggest that patterns of IQ–DLS discrepancies observed in prior studies may not fully reflect the developmental experiences of autistic individuals from more socioeconomically and racially diverse backgrounds.

The use of binary indicators (e.g., employed/unemployed, living independently/living with support) for many of the adult milestones examined here does not capture the true variability of autistic adult experiences and may have contributed to the lack of associations seen between IQ‐DLS discrepancy trajectories and adult experiences. However, there were also null findings for associations between trajectory groups and adult well‐being, which was measured via a continuous variable. Categorical variables measuring friendship and living status were investigated, but the bimodal outcome distributions observed in this sample limited the utility of this approach. For example, most participants either lived in the family home or independently—few individuals lived with part‐time support or some other intermediary arrangement. It is also possible that the lack of significant differences in child and adult characteristics across trajectory groups observed in the current sample is due to the relatively small sample size within the identified trajectory groups. Additional work in larger samples is needed to better understand how IQ‐DLS discrepancies relate to demographic and phenotypic characteristics across the life course.

The Vineland Adaptive Behavior Scales is not an ideal measure, particularly for quantifying DLS in adulthood. The DLS items on the Vineland are heavily weighted toward skills that are developmentally salient in childhood and adolescence and emphasize whether the person *does* the task without reminders or support, regardless of their *ability to* do the task. Additionally, VABS AEs become sparser with increasing age, and the maximum VABS AE is 22 years. As a result, a 1‐point difference in raw scores in the Personal subdomain on the VABS‐II can result in a 3+ year difference in AEs, which may overemphasize discrepancies at higher skill levels. There is some evidence that the Adaptive Behavior Assessment Scale (ABAS; Harrison and Oakland 2015) is a more valid and reliable measure of adaptive behavior for autistic adults than the VABS (Glover et al. 2022). However, little work to date has explored how ABAS scores relate to concurrent or later outcomes for autistic adults. Additional work is also needed to clarify whether the most recent edition of the Vineland (VABS‐3; Sparrow et al. 2016) would result in similar trajectories to those presented here, which used data from the first and second editions of the VABS.

## 6 Conclusion

This study is one of the first longitudinal examinations of IQ‐DLS discrepancies in autistic individuals with average or better cognitive abilities. Nearly 60% of participants had commensurate cognitive abilities and DLS (IQ = DLS). The subset of participants with stronger cognitive abilities than DLS (IQ > DLS) was significantly more likely to be white; participant race was the only characteristic from early childhood that predicted IQ‐DLS trajectory group inclusion. Analyses of DLS domain standard scores and Personal, Domestic, and Community subdomain AEs illustrated very similar patterns, suggesting DLS development is consistent across DLS subtypes in autistic individuals with average or better IQ. Surprisingly, no significant associations were found between IQ‐DLS trajectories and adult experiences, although further work in larger samples is needed to confirm this. Future work should also examine how opportunities to learn and practice DLS may vary for autistic individuals, particularly across diverse racial/ethnic and socioeconomic backgrounds.

## Funding

This work was supported by the National Institute on Aging, R01‐AG080599; Autism Science Foundation.

## Conflicts of Interest

The authors declare no conflicts of interest.

## Supporting information


**Table S1:** Full scale IQ and DLS standard scores by DLS domain trajectory groups.
**Table S2:** NVMA and DLS AE by personal subdomain trajectory groups.
**Table S3:** NVMA and DLS AE by domestic subdomain trajectory groups.
**Table S4:** NVMA and DLS AE by community subdomain trajectory groups.
**Table S5:** DLS domain (standard scores) and subdomain (age equivalents) discrepancy trajectories model selection in autistic participants only (*n* = 65).
**Figure S1:** Trajectories of IQ—DLS standard score discrepancies from ages 2–25 in autistic participants only.
**Figure S2:** Trajectories of NV abilities—DLS AE discrepancy scores from ages 2–25 in autistic participants only.

## Data Availability

The data that support the findings of this study are available from the corresponding author upon reasonable request.
