# Supplementary material for: Limited Discrepancy Between Cognitive Ability and Daily Living Skills in Autism: A Longitudinal Study From Ages 2–25
Source: Autism Res. 2026 Jun 2;19(7):e70280. doi: 10.1002/aur.70280 (PMC13377276; doi:10.1002/aur.70280)
Supplement: Supplementary file 1 — Table S1: Full scale IQ and DLS standard scores by DLS domain trajectory groups. Table S2: NVMA and DLS AE by personal subdomain trajectory groups. Table S3: NVMA and DLS AE by domestic subdomain trajectory groups. Table S4: NVMA and DLS AE by community subdomain trajectory groups. Table S5: DLS domain (standard scores) and subdomain (age equivalents) discrepancy trajectories model selection in autistic participants only (n = 65). Figure S1: Trajectories of IQ—DLS standard score discrepancies from ages 2–25 in autistic participants only. Figure S2: Trajectories of NV abilities—DLS AE discrepancy scores from ages 2–25 in autistic participants only. [file AUR-19-0-s001.docx]

**Supplement 1.**

|  |  | **Data Collection Timepoint** | | | | | |
| --- | --- | --- | --- | --- | --- | --- | --- |
| **n** | | **2** | **3** | **5** | **9** | **18** | **25** |
|  |  | 73 | 60 | 45 | 58 | 54 | 30 |
|  |  | **m (SD)**  **[range]** | | | | | |
| **IQ>DLS** | **Full-Scale IQ** | 77.20 (12.87)  [52.0-103.5] | 86.27 (13.53)  [58.0-113.0] | 92.11 (17.50)  [65.5-128.0] | 104.98 (18.64)  [64.0-139.0] | 117.33 (13.61)  [84.0-130.0] | 120.0 (12.90)  [100.0-130.0] |
|  | **DLS Standard Score** | 72.31 (8.79)  [59.0-101.0] | 71.17 (9.76)  [57.0-94.0] | 77.44 (11.64)  [62.0-100.0] | 73.62 (22.14)  [20.0-108.0] | 77.40 (15.56)  [57.0-102.0] | 74.60 (13.43)  [59.0-95.0] |
| **IQ=DLS** | **Full-Scale IQ** | 75.09 (17.38)  [39.5-115.0] | 77.39 (14.10)  [45.0-113.0] | 83.91 (13.0)  [56.5-109.5] | 91.60 (17.37)  [58.0-126.0] | 100.89 (15.49)  [74.0-129.0] | 97.40 (22.19)  [69.0-117.0] |
|  | **DLS Standard Score** | 74.93 (10.23)  [60.0-99.0] | 74.08 (11.13)  [59.0-103.0] | 84.48 (16.01)  [63.0-123.0] | 86.06 (22.24)  [19.0-119.0] | 86.11 (13.52)  [61.0-112.0] | 82.40 (19.39)  [65.0-112.0] |

Table S1. Full Scale IQ and DLS Standard Scores by DLS Domain Trajectory Groups

Table S2. NVMA and DLS AE by Personal Subdomain Trajectory Groups

|  |  | **Data Collection Timepoint** | | | | | |
| --- | --- | --- | --- | --- | --- | --- | --- |
| **n** | | **2** | **3** | **5** | **9** | **18** | **25** |
|  |  | 73 | 60 | 45 | 58 | 54 | 30 |
|  |  | **m (SD) [range]** | | | | | |
| **IQ>DLS** | **NV Abilities** | 2.23 (0.66) [1.50–3.92] | 3.39 (1.39) [2.31–8.17] | 4.49 (1.56) [2.80–8.19] | 10.08 (2.11) [6.08–13.84] | 30.38 (4.02) [26.31–40.01] | 28.56 (3.50) [24.80–33.25] |
|  | **Personal AE** | 1.60 (0.32) [1.17–2.50] | 2.55 (0.72) [1.75–4.25] | 3.44 (0.99) [2.42–6.08] | 6.51 (1.71) [3.58–10.17] | 15.28 (3.12) [10.50–20.00] | 17.31 (4.37) [11.50–22.00] |
| **IQ=DLS** | **NV Abilities** | 1.93 (0.44) [1.16–3.17] | 3.05 (0.69) [1.87–4.68] | 4.17 (1.03) [2.25–6.29] | 8.49 (1.97) [4.31–13.76] | 25.02 (4.44) [18.32–32.47] | 20.49 (3.97) [16.18–25.96] |
|  | **Personal AE** | 1.58 (0.50) [0.67–2.83] | 2.45 (0.57) [1.58–3.58] | 3.68 (1.02) [2.33–6.33] | 7.38 (2.59) [3.17–13.50] | 16.67 (2.48) [10.50–20.00] | 15.00 (2.48) [11.50–18.50] |

Note. All scores are presented in years.

|  |  | **Data Collection Timepoint** | | | | | |
| --- | --- | --- | --- | --- | --- | --- | --- |
| **n** | | **2** | **3** | **5** | **9** | **18** | **25** |
|  |  | 73 | 60 | 45 | 58 | 54 | 30 |
|  |  | **m (SD)**  **[range]** | | | | | |
| **IQ>DLS** | **NV Abilities** | 2.22 (0.64) [1.42–3.92] | 3.36 (1.35) [1.97–8.17] | 4.42 (1.52) [2.80–8.19] | 9.95 (2.28) [5.67–13.84] | 21.03 (3.66) [13.32–30.37] | 28.31 (5.38) [18.32–40.01] |
|  | **Domestic AE** | 1.49 (0.27) [1.33–2.00] | 2.15 (0.68) [1.33–4.42] | 3.57 (1.23) [1.75–6.83] | 6.78 (1.29) [2.75–8.42] | 13.70 (5.02) [2.92–22.00] | 14.41 (4.59) [8.50–20.00] |
| **IQ=DLS** | **NV Abilities** | 1.92 (0.44) [1.16–3.17] | 3.05 (0.68) [1.87–4.68] | 4.20 (1.04) [2.25–6.29] | 8.53 (1.90) [4.31–13.76] | 18.49 (3.38) [12.25–24.79] | 25.65 (4.42) [18.43–32.47] |
|  | **Domestic AE** | 1.57 (0.37) [1.33–3.00] | 2.22 (0.65) [1.33–4.08] | 3.97 (1.17) [2.00–6.42] | 7.86 (2.25) [3.00–11.75] | 17.18 (4.00) [7.42–22.00] | 16.19 (3.74) [11.50–22.00] |

Note. All scores are presented in years.

Table S3. NVMA and DLS AE by Domestic Subdomain Trajectory Groups

|  |  | **Data Collection Timepoint** | | | | | |
| --- | --- | --- | --- | --- | --- | --- | --- |
| **n** | | **2** | **3** | **5** | **9** | **18** | **25** |
|  |  | 73 | 60 | 45 | 58 | 54 | 30 |
|  |  | **m (SD)[range]** | | | | | |
| **IQ>DLS** | **NV Abilities** | 2.24 (0.62) [1.42–3.92] | 3.52 (1.34) [1.97–8.17] | 4.52 (1.57) [2.84–8.19] | 10.03 (2.23) [5.67–13.84] | 21.04 (3.65) [13.32–30.37] | 27.77 (5.41) [18.32–40.01] |
|  | **Community AE** | 1.19 (0.34) [0.42–1.42] | 2.07 (0.74) [1.42–3.50] | 3.13 (0.98) [2.17–5.75] | 8.21 (1.60) [4.75–10.33] | 13.75 (3.79) [6.08–19.00] | 16.41 (3.83) [8.42–19.00] |
| **IQ=DLS** | **NV Abilities** | 1.91 (0.44) [1.16–3.17] | 2.97 (0.63) [1.87–4.35] | 4.17 (1.04) [2.25–6.29] | 8.48 (1.89) [4.31–13.76] | 17.99 (3.08) [12.25–23.30] | 25.84 (4.42) [18.43–32.47] |
|  | **Community AE** | 1.18 (0.60) [0.42–2.67] | 1.98 (0.59) [1.00–3.50] | 3.55 (1.01) [1.75–6.25] | 8.31 (2.98) [2.92–14.75] | 15.89 (3.11) [8.00–20.00] | 18.44 (2.42) [14.00–21.00] |

Note. All scores are presented in years.

Table S4. NVMA and DLS AE by Community Subdomain Trajectory Groups

Table S5. DLS Domain (Standard Scores) and Subdomain (Age Equivalents) Discrepancy Trajectories Model Selection in Autistic Participants Only (n = 65)

|  |  | **DLS Domain**  **Standard Scores** | | | | | **Personal Subdomain**  **Age Equivalents** | | | |
| --- | --- | --- | --- | --- | --- | --- | --- | --- | --- | --- |
|  | **Model** | BIC | AIC | Smallest Group % | | BIC | | AIC | Smallest Group % |  |
|  | 1 Class Model | -1414.76 | -1409.10 | — | | -1510.34 | | -1504.80 | — |  |
|  | 2 Class Model | **-1395.14** | **-1383.82** | **43.59** | | **-1483.87** | | **-1472.79** | **37.62** |  |
|  | 3 Class Model | -1392.37 | -1375.39 | 5.0 | | -1492.41 | | -1475.79 | 0.00 |  |
|  |  | **Domestic Subdomain**  **Age Equivalents** | | | **Community Subdomain**  **Age Equivalents** | | | | | |
|  | **Model** | BIC | AIC | Smallest Group % | | BIC | | AIC | Smallest Group % |  |
|  | 1 Class Model | -1479.34 | -1477.10 | — | | -1435.34 | | -1429.83 | — |  |
|  | 2 Class Model | **-1438.37** | **-1427.37** | **36.36** | | **-1378.73** | | **-1367.71** | **42.23** |  |
|  | 3 Class Model | -1446.87 | -1430.37 | 0.00 | | -1383.46 | | -1366.93 | 1.54 |  |

Note: Final model selected in bold.

Figure S1. Trajectories of IQ – DLS Standard Score Discrepancies from Ages 2-25 in Autistic Participants Only


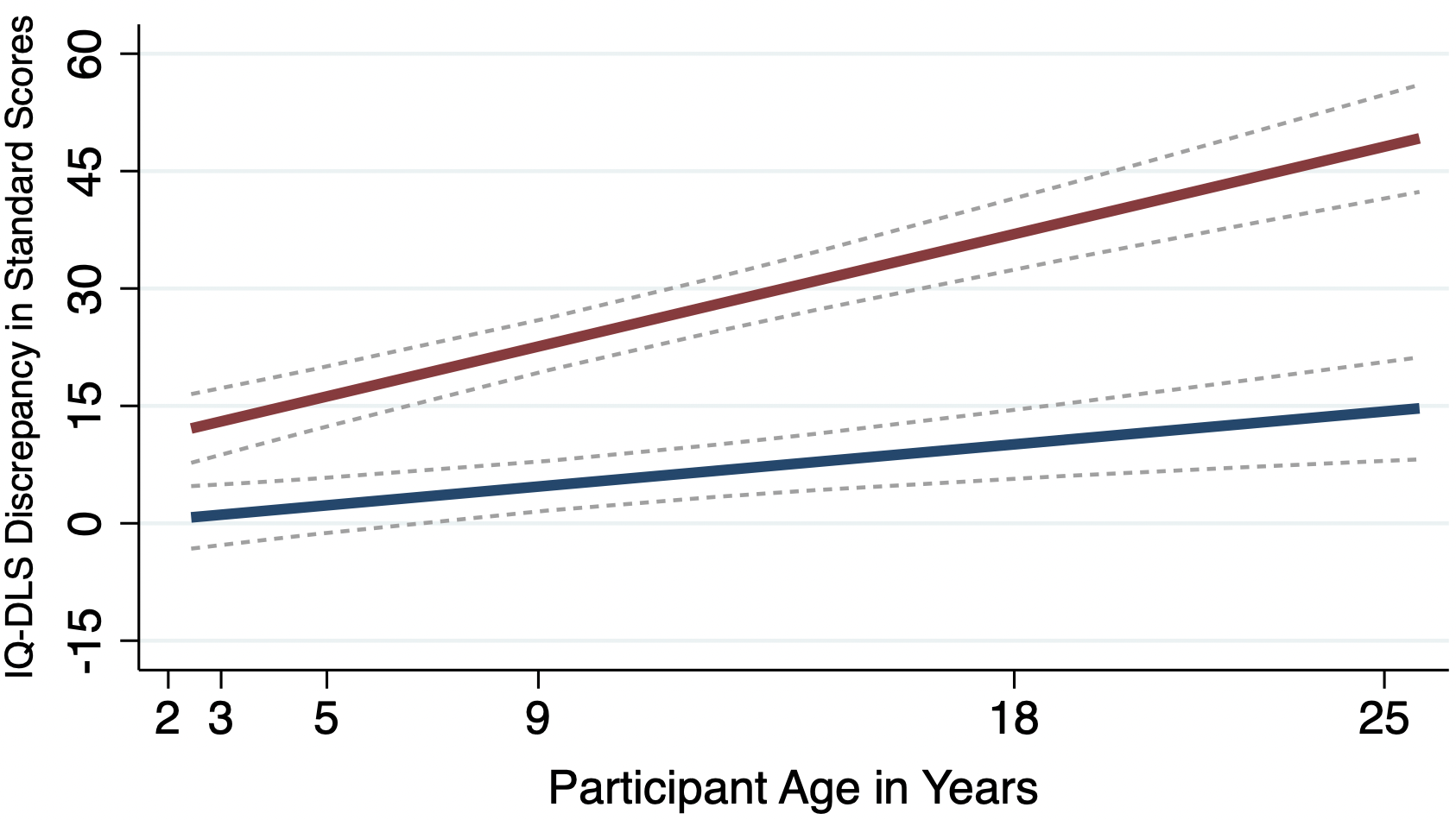


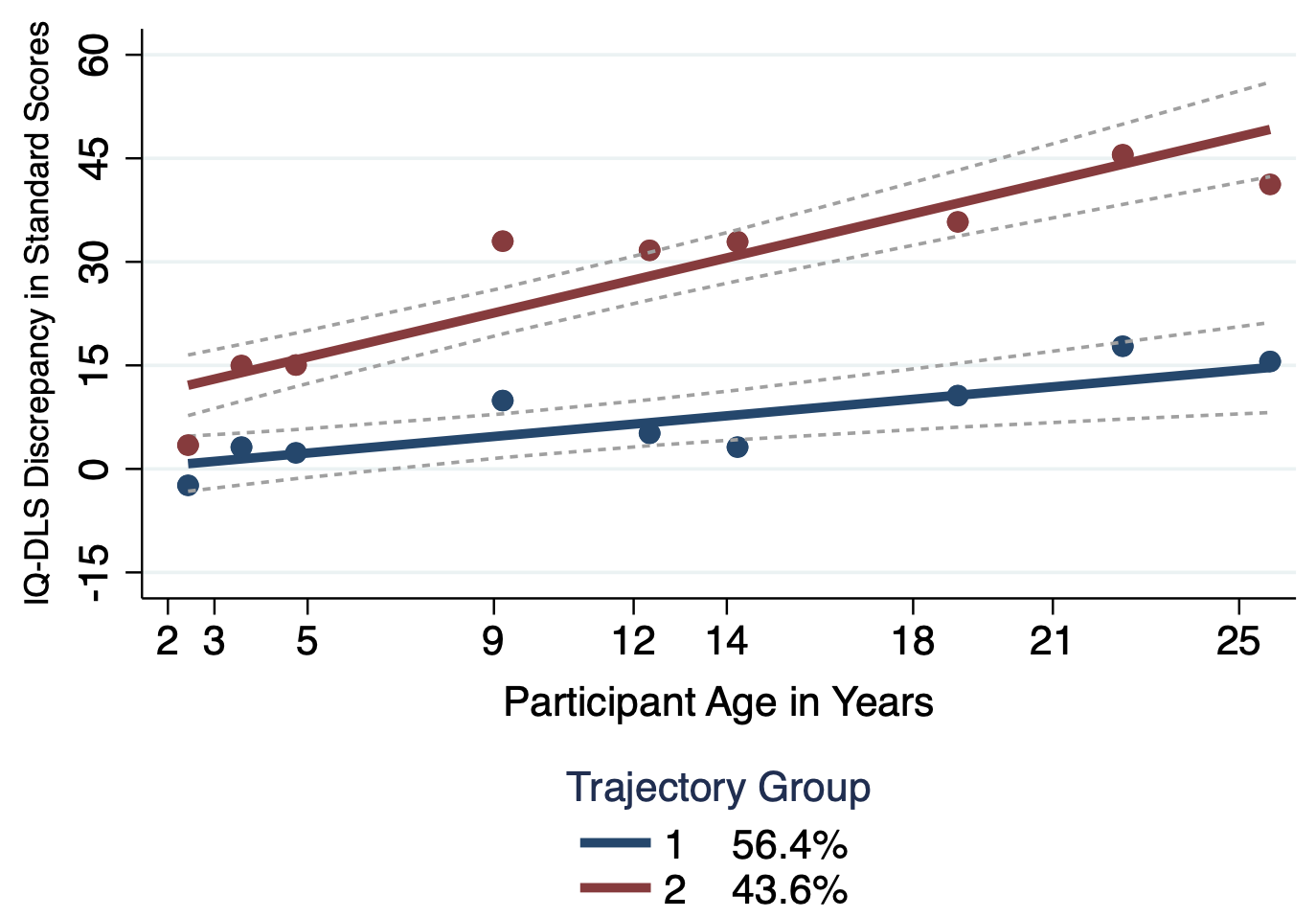


Figure S2. Trajectories of NV Abilities – DLS AE Discrepancy Scores from Ages 2-25 in Autistic Participants Only


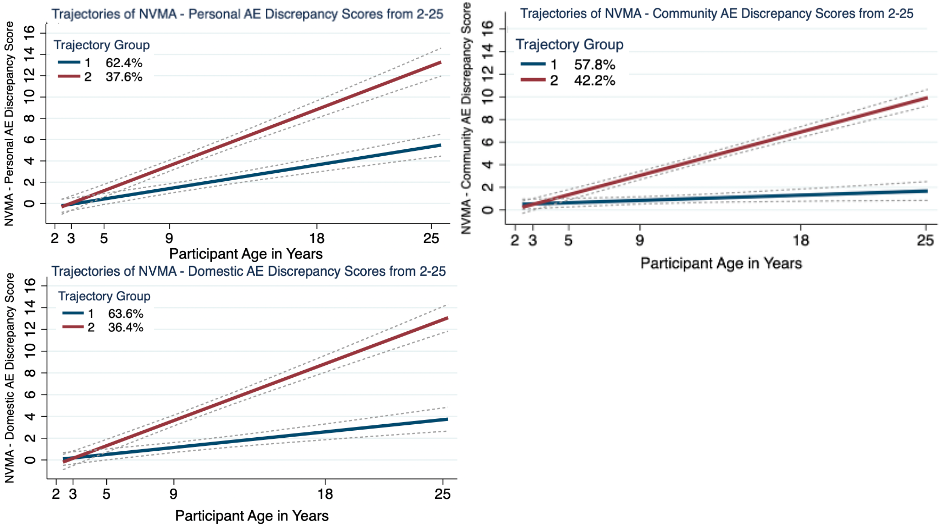


Note. AE discrepancy scores are listed in years.
